# Differential protein expression profile in the hypothalamic GT1-7 cell line after exposure to anabolic androgenic steroids

**DOI:** 10.1371/journal.pone.0180409

**Published:** 2017-07-18

**Authors:** Freddyson J. Martínez-Rivera, Juliana Pérez-Laspiur, María E. Santiago-Gascot, Abner G. Alemán-Reyes, Emanuel García-Santiago, Yolanda Rodríguez-Pérez, Cristhian Calo-Guadalupe, Inelia Otero-Pagán, Roxsana N. Ayala-Pagán, Magdiel Martínez, Yisel M. Cantres-Rosario, Loyda M. Meléndez, Jennifer L. Barreto-Estrada

**Affiliations:** 1 Department of Anatomy and Neurobiology, Medical Sciences Campus, University of Puerto Rico, San Juan, Puerto Rico, United States of America; 2 Translational Proteomics Center-RCMI, Medical Sciences Campus, University of Puerto Rico, San Juan, Puerto Rico, United States of America; 3 Department of Biology, University of Puerto Rico, Río Piedras Campus, San Juan, Puerto Rico, United States of America; 4 Department of Biotechnology, Universidad del Este, Carolina, Puerto Rico, United States of America; 5 Department of Physiology and Biophysics, Medical Sciences Campus, University of Puerto Rico, San Juan, Puerto Rico, United States of America; 6 Department of Microbiology and Medical Zoology, Medical Sciences Campus, University of Puerto Rico, San Juan, Puerto Rico, United States of America; Universite de Rouen, FRANCE

## Abstract

The abuse of anabolic androgenic steroids (AAS) has been considered a major public health problem during decades. Supraphysiological doses of AAS may lead to a variety of neuroendocrine problems. Precisely, the hypothalamic-pituitary-gonadal (HPG) axis is one of the body systems that is mainly influenced by steroidal hormones. Fluctuations of the hormonal milieu result in alterations of reproductive function, which are made through changes in hypothalamic neurons expressing gonadotropin-releasing hormone (GnRH). In fact, previous studies have shown that AAS modulate the activity of these neurons through steroid-sensitive afferents. To increase knowledge about the cellular mechanisms induced by AAS in GnRH neurons, we performed proteomic analyses of the murine hypothalamic GT1-7 cell line after exposure to 17α-methyltestosterone (17α-meT; 1 μM). These cells represent a good model for studying regulatory processes because they exhibit the typical characteristics of GnRH neurons, and respond to compounds that modulate GnRH *in vivo*. Two-dimensional difference in gel electrophoresis (2D-DIGE) and mass spectrometry analyses identified a total of 17 different proteins that were significantly affected by supraphysiological levels of AAS. Furthermore, pathway analyses showed that modulated proteins were mainly associated to glucose metabolism, drug detoxification, stress response and cell cycle. Validation of many of these proteins, such as GSTM1, ERH, GAPDH, PEBP1 and PDIA6, were confirmed by western blotting. We further demonstrated that AAS exposure decreased expression of estrogen receptors and GnRH, while two important signaling pathway proteins p-ERK, and p-p38, were modulated. Our results suggest that steroids have the capacity to directly affect the neuroendocrine system by modulating key cellular processes for the control of reproductive function.

## Introduction

The anabolic androgenic steroids (AAS) are synthetic derivatives of testosterone, created to provide anabolic potency and low androgenic effect [1]. While most users abuse AAS to improve physical performance, nearly all of them are unaware of the numerous side effects that can be associated with androgen misuse [2]. Cardiovascular diseases, cancer, liver dysfunction and psychiatric disorders are some of those well- documented problems [2,3]. Similarly, under the influence of AAS, the hypothalamic-pituitary-gonadal (HPG) axis is one of the most affected body systems [4,5]. The neural control of this axis resides within the hypothalamus, which is characterized by the expression of high levels of androgen and estrogen receptors (AR and ER) [6]. The medial preoptic area (mPOA) is a steroid-sensitive hypothalamic region, that is populated by neurons expressing gonadotropin-releasing hormone (GnRH) [7]. Indeed, GnRH neurons respond to fluctuations of gonadal steroids, which result in neuroplasticity changes leading to pulsatile secretion of this peptide. *In vivo* and *in vitro* studies have shown that exposure to steroids is associated with alterations on GnRH expression and secretion. For instance, studies in rodents [8], and cells [9,10] showed that exposure to either androgens or estrogens reduced GnRH transcripts.

To date, few studies have investigated the effect of AAS in GnRH neurons. In this regard, electrophysiological studies have shown that AAS modulate the activity of these cells [4,5]. For example, the AAS 17α-methyltestosterone (17α-meT; 7.5 mg/kg), increased presynaptic GABA_A_ receptor (GABA_A_R) currents of mPOA steroid-sensitive neurons, resulting in inhibition of GnRH cells [4]. Similarly, female mice exposed to 17 α-meT (7.5 mg/kg), displayed a diestrous-like pattern activity in GnRH neurons through the suppression of presynaptic kisspeptin excitatory inputs from the anteroventral periventricular nucleus [5]. Although these studies demonstrated neuroendocrine modulation by AAS, there is still a lack of a complete protein profile on GnRH neurosecretory cells after AAS exposure, which has the potential to reveal specific alterations in regulatory processes of the reproductive axis. In this study, we characterized the proteomic profile of GnRH neurons after exposure to supraphysiological levels of 17 α-meT.

Given the scattered concentration of GnRH neurosecretory cells within the mPOA [11], and the difficulty to establish an *in vivo* approach to study these neurons [9], we used the murine immortalized cell line of GnRH-secreting hypothalamic neurons (GT1-7) [12]. Certainly, GT1-7 cells have been very useful in studying regulatory processes because they exhibit the typical characteristics of GnRH neurons, and respond to the same compounds that modulate GnRH secretion *in vivo* [13–15]. Moreover, evidence reveals that GT1-7 cells express AR [16,17], ER [18,19], and receptors for GABA (GABA_A_R) [20], cellular properties that confer responsiveness to androgenic and estrogenic compounds.

The use of omics technologies has gained popularity to uncover the use of anabolic agents in human sports, animal husbandry [21], and aquaculture [22]. Indeed, proteomic analyses have been applied for the screening of steroid effects on body tissues such as prostate [23], gonads [22], breast [24], muscles [25] and blood components [26]. In this study, we used two-dimensional difference in gel electrophoresis (2D-DIGE) in combination with mass spectrometry and western blotting to profile the proteome of GnRH neurons. We hypothesized that 17α-meT will modulate the expression of proteins associated with neuroendocrine regulation, synaptic plasticity, and cellular stress; key biological processes that might impact reproductive competence and integrity.

## Materials and methods

### Cell culture and reagents

GT1-7 cells were grown as previously described [12]. In brief, cells were maintained in DMEM (Mediatech, Manassas, VA) supplemented with 10% fetal bovine serum (FBS; Hyclone, Waltham, MA) and penicillin/streptomycin (Gibco, Grand Island, NY). Cells were grown in 25 cm culture flasks and maintained in a humidified incubator at 37°C and 5% CO_2_. Each culture flask represents an independent biological replicate.

### Drug

A 2D-DIGE experiment was performed using four independent biological replicates per treatment (Control: n = 4; AAS: n = 4). Before AAS exposure, cells were grown in steroid-free serum (Hyclone Waltham, MA) during the log-phase growth (70–80% confluency). Thereafter, four (4) samples were treated with a supraphysiological dose of the AAS, 17α-methyltestosterone (17α-meT: 1 μM; Sigma, St. Louis, MO) for 48 h as previously described [27]. Control samples were treated with vehicle (30% cyclodextrin in 0.9% saline; Sigma, St. Louis, MO). 17α-meT was chosen as the presence of the C17 methyl group reduces its aromatization to 17β-estradiol [28], and inhibits aromatization [29,30]. As the normal level of testosterone in male serum is 1 X 10^−8^ M (0.01 μM) [31,32], the regimen used in our experiment (1 μM for 48 h) [27] reflects a chronic supraphysiological dose. Cell viability after AAS exposure was assessed by trypan blue exclusion assay as previously described with some modifications [33].

### Caspase activity

Caspase 3 activity was measured using the Caspase-Glo^®^ 3/7 (Promega Co., Madison, WI), in which 50,000 cells/well were initially seeded in a flat bottom 96-well plate. Cells were incubated with equal volumes of the reagent to the culture medium for 1 h, and a Varioskan Flash Reader (Thermo Fisher Scientific, Waltham, MA) was used to assess relative luminescence. Caspase 9 activity was measured using the Caspase-9 Fluorometric assay (R&D Systems, Inc. Minneapolis, MN). Each sample contained the cell lysate (100 μg of protein in 50 μL), 50 μL of 2X reaction buffer, and 5 μL of caspase 9 fluorometric substrate (LEHD-AFC). The reaction was incubated for 1 h at 37°C in a flat bottom 96-well microplate. Fluorescence signal was indicative of caspase activation, and it was measured on a fluorescent plate reader (Gemini SpectraMax, Molecular Devices, CA, USA).

### Proteomic analyses

#### Protein extraction and protein quantification

Cells were harvested after 48 h of AAS exposure. Lysis Buffer (7 M urea, 2 M thiourea, 4% CHAPS; GE Healthcare, Pittsburgh, PA) and a cocktail of protease inhibitors (BioVision, Inc., Milpitas, CA) were added to the cell pellets. Samples were centrifuged at 13,000 g for 15 min at 4°C. Supernatants containing the cellular protein fractions were recovered and protein extracts were purified by precipitation, using a 2-D Clean-Up Kit (GE Healthcare, Pittsburgh, PA). After precipitation, protein pellets were resuspended in cell lysis buffer (30 mM Tris-HCL pH = 8, 7 M urea, 2 M thiourea, 4% CHAPS). Protein concentration was measured using a 2-D Quant Kit (GE Healthcare, Pittsburgh, PA). Kits were used as suggested by manufacturer’s instructions.

#### Sample labeling

Cy5 and Cy3 labeling for analytical gels: Five (5) μg of total protein from GT1-7 cells samples (4 controls, 4 AAS) were speed-vacuum dried and resuspended in cell lysis buffer. Samples were reduced by incubation with 2 nM TCEP (tris-[2-carboxyethyl] phosphoine hydrochloride; GE Healthcare, Pittsburgh, PA) at 37°C for 1 h and then labeled with 4 nM of Cy5 saturation dye (GE Healthcare, Pittsburgh, PA) for 30 min at 37°C. One volume sample buffer (7 M urea, 2 M thiourea, 4% CHAPS), pH3-10NL IPG buffer, and 130 mM DTT was added to the samples. An internal standard was prepared by pooling 5 μg total protein of each sample, mixed together, and then speed vacuum-dried. Protein pellet was resuspended with cell lysis buffer and reduced with 2nM TCEP per 5 μg protein for 1 h at 37°C. The proteins were labeled with 4 nM of Cy3 saturation dye per 5 μg protein for 30 min at 37°C. One volume sample buffer was added to the samples. Each 5 μg of sample labeled with Cy5 was mixed with 5 μg of internal standard labeled with Cy3. Rehydration buffer (7 M urea, 2 M thiourea, 2% CHAPS), and 60 mM DTT, pH 3-10NL IPG buffer was added to a final volume of 450 μl.

Cy3 labeling for preparative gels: A total of 250 μg of protein (31.25 μg/ sample) from samples represented in analytical gels were pooled, speed vacuum-dried, resuspended in 250 μl of cell lysis buffer and treated with TCEP for 1 h at 37°C. Protein (250 μg) was labeled with Cy3 saturation dye for 30 min at 37°C. To stop the reaction, sample buffer was added to a final volume of 450 μl.

#### 2D-DIGE

First dimension was carried out with an Ettan IPGphor apparatus (GE Healthcare, Pittsburgh, PA). Samples were loaded on 24 cm long Immobiline DryStrips gels with non-linear immobilized pH gradient 3–10 by overnight rehydration. Each gel contained the internal standard and one of the samples (analytical gels, after saturation labeling) or pooled material in the case of the preparative gel strip. Isoelectric focusing was carried out at a constant temperature of 20°C with a total of 78.5 kVh. Before the second dimension, separation strips were incubated with an equilibration solution (50 mM Tris-HCL pH = 8.8, 6 M urea, 30% glycerol, 2% SDS, 0.01 bromophenol blue containing 100 mM DTT) for 15 min. Then, the strips were loaded on the top of pre-cast 12% polyacrylamide gels and fixed with 0.5% agarose. The second dimension was carried out with an Ettan DALTtwelve Electrophoresis System (GE Healthcare, Pittsburgh, PA) at 20°C. Current was held constant to 12 mA per gel for an overnight period. On the next day, current was increased to 20 mA per gel until bromophenol blue mark the end of the gel. For visualization of protein spots, signals were collected at excitation wavelength for Cy3 and Cy5 labeled sample at 540 and 635 nm, respectively, using Ettan DIGE Imager (GE Healthcare, Pittsburgh, PA). Gels were scanned at 100 μm resolution and analyzed using DeCyder 2-D 6.5 software (GE Healthcare, Pittsburgh, PA). Using a 2 mm diameter tip, spots selected for protein identification after DeCyder statistical analysis (P<0.05) were picked from the preparative gel by automatic Ettan Spot Picker (GE Healthcare, Pittsburgh, PA).

#### In-gel digestion

Spots picked from 2D-DIGE preparative gel were washed at RT with 200 μl of 50% acetonitrile and 50 mM ammonium bicarbonate solution for 1 h. Gel pieces were dried and then incubated with trypsin overnight digestion at 37°C. Resulting peptides were extracted using a mixture of 60% acetonitrile and 0.1% trifluoroacetic acid (TFA). Samples were dried and resuspended in 0.5% trifluoroacetic acid. All samples were desalted and purified using C18 ZipTips (EMD Millipore, Billerica, MA) according to manufacturer’s recommendations and resuspended in 2% acetonitrile with 0.1% formic acid prior to liquid chromatography tandem mass spectrometry (LC-MS/MS) analysis.

#### Mass spectrometry and protein identification

Tryptic peptides were reconstituted in 40 μL of 0.1% TFA (v/v) in water: acetonitrile (95:5) and 4 μL was directly loaded at 4 μL/min for 7 min onto a custom-made trap column (100 μm I.D. fused silica with Kasil frit) containing 2 cm of 200Å, 5 μm Magic C18AQ particles (Bruker-Michrom, Auburn, CA). Peptides were eluted using a custom-made analytical column (75 μm I.D. fused silica) with gravity-pulled tip and packed with 25 cm 100Å, 5 μm Magic C18AQ particles (Bruker-Michrom, Auburn, CA). Peptides were eluted with a linear gradient from 100% solvent A (0.1% formic acid:acetonitrile [95:05]) to 35% solvent B (acetonitrile containing 0.1% formic acid) in 30 min at 300 nL/min, using a Proxeon Easy nanoLC system directly coupled to a LTQ Orbitrap Velos mass spectrometer (Thermo Scientific, Waltham, MA). Data were acquired using a data-dependent acquisition routine of acquiring one mass spectrum from *m/z* 350–2000 in the Orbitrap (resolution 60,000) followed by tandem mass spectrometry scans in the LTQ linear ion trap of the 10 most abundant precursor ions found in the mass spectrum. Charge state rejection of singly charged ions and dynamic exclusion was utilized to minimize data redundancy and maximize peptide identification. The raw data files were processed into peak lists for database searching.

Database Searching of all MS/MS samples was performed using Mascot (Matrix Science, London, UK; version 2.3.02) and X! Tandem (The GPM, thegpm.org; version CYCLONE [2010.12.01.1]). Charge state deconvolution and deisotoping were not performed. X! Tandem was set up to search a subset of the SwissProt_012512 database and Mascot was set up to search the SwissProt_022212 database (selected for Mus., unknown version, 16531 entries) assuming the digestion enzyme trypsin. Mascot and X! Tandem were searched with a fragment ion mass tolerance of 0.50 Da and a parent ion tolerance of 15 PPM. Pyro-glu from E of the n-terminus, oxidation of methionine, acetylation of the n-terminus and iodoacetamide derivative of cysteine were specified in X! Tandem as variable modifications. Pyro-glu from E of the n terminus, oxidation of methionine, acetylation of the n-terminus, iodoacetamide derivative of cysteine and acrylamide adduct of cysteine were specified in Mascot as variable modifications.

Scaffold (version Scaffold_3.4.5, Proteome Software Inc., Portland, OR) was used to validate MS/MS based peptide and protein identifications. Peptide identifications were accepted if they could be established at greater than 80% probability, as specified by the Peptide Prophet algorithm [34]. Protein identifications were accepted if they could be established at greater than 90% probability and contained at least 2 identified peptides. Protein probabilities were assigned by the Protein Prophet algorithm [35]. Proteins that contained similar peptides and could not be differentiated based on MS/MS analysis alone were grouped to satisfy the principles of parsimony.

### Networks and pathways analysis

Uniprot database (www.uniprot.org) was used to identify protein localization and function. Ingenuity pathways analysis (IPA) knowledge base (www.ingenuity.com) was used to identify predominant interaction networks and biological functions of differentially expressed proteins [36]. This software uses computational algorithms to identify networks consisting of proteins of interest and their interactions with other proteins in the knowledge base. The network scores (negative log of the P value) are calculated according to the fit of the network to the focus proteins. In addition, IPA knowledge base identifies global function and canonical pathways of the entire data set of proteins. The significance values for the canonical pathways are calculated by Fisher's exact test right-tailed. Specifically, this test compares the number of proteins that contribute in a given pathway or process, relative to the total number of occurrences of these proteins in all pathway annotations stored in the IPA knowledge base.

### Validation of differential protein expression

#### Sample preparation

GT1-7 cells were grown as previously described in proteomic experiments. Cells were washed with ice-cold PBS, and lysed with CelLytic Mammalian Lysis/Extraction Reagent, supplemented with SIGMAFAST^™^ Protease Inhibitor Tablets (Sigma–Aldrich, MO). Extracts were maintained with constant agitation for 30 min at 4°C and then centrifuged for 20 min at 17, 000 g. Supernatants were collected and used to determine total protein concentration using the Bradford Quick-Start Protein Assay (Bio-Rad Laboratories, Hercules, CA). All procedures were performed at 4°C and samples were stored at -80°C for further protein extraction and western blotting.

#### Western blotting

Western blotting was performed as previously described [37], with some modifications. Equal amounts of whole protein extracts were suspended in 6x Laemmli Sample Buffer, heated, loaded, and then electrophoresed on Pre-Casted TGX-SDS gels (Bio-Rad, CA). Gels were transferred to nitrocellulose membranes using the Bio-Rad Turbo Trans-Blot apparatus. Membranes were blocked with 5% (w/v) non-fat milk in TBST [25 mM Tris–HCl, pH 7.4, 150 mM NaCl and 0.1% (v/v) Tween-20] overnight at 4°C and the appropriate primary antibodies were added overnight at 4°C. Membranes were then washed with TBST and probed with the corresponding horseradish peroxidase-conjugated (HRP) secondary antibodies at 22°C for 1 h. Blots were visualized using an enhanced chemiluminescence kit (SuperSignal Femto, Pierce, IL) and images were obtained using a VersaDoc 1000 system (Bio-Rad, CA). All Western blots were performed at least in triplicate from three independent experiments, and densitometry analyses were normalized to β-actin expression using NIH ImageJ (v1.47d). Data from Western blots is presented as mean ± standard error of the mean (S.E.M.). Student’s t-test analyses were employed and statistical significance was established as *P ≤ 0.05; **P ≤ 0.01.

#### Antibodies

The following primary antibodies were used: mouse anti-ERH (1:1000), mouse anti-PDIA6/ERP (1:1000), rabbit anti-ERα (1:1500), goat anti-AR (1:1000) and rabbit anti-GnRH1 (1:1000) from Santa Cruz Biotechnology (SCBT), Dallas, TX; rabbit anti-GSTM1 (1:2000) from Thermo Scientific, Waltham, MA; rabbit anti-PEBP1/RKIP (1:1000), rabbit anti-GAPDH (G3P)-HRP conjugated (1:3000), rabbit anti-p-ERK (1:10000), rabbit anti-ERK (1:6000), rabbit anti-p-p38 MAPK (Thr180/Tyr182) (1:10000), rabbit anti-AKT (pan) (1:15000) and rabbit anti-β-actin HRP conjugated (1:3000) from Cell Signaling Technology (CST) Danvers, MA.

## Results

### Proteomic changes in GT1-7 cells after AAS treatment

To investigate the effect of a supraphysiological dose of AAS in the proteome of GT1-7 cells, we used 2D-DIGE analyses in extracts from vehicle and 17α-meT treated cells. Before examining the proteome, we first demonstrated that 17α-meT at 1 μM did not affect cell viability at 48 h (Control: 92.07 ± 0.012; 17α-meT: 92.82 ± 0.013; p = 0.703, unpaired t-test). Likewise, there were no AAS-induced apoptosis as indicated by caspase activity assays. Values of relative luminescence units for caspase 3 were: Control: 6.5 X 10^6^ ± 2.99 X 10^5^; 17α-meT: 6.99 X 10^6^ ± 1.29 X 10^5^; t_6_ = 1.527; p = 0.177; unpaired t-test. Values of relative fluorescence units for caspase 9 were: Control: 468.21 ± 31.54; 17α-meT: 464.61 ± 22.02; t_5_ = 0.097; p = 0.926, unpaired t-test.

**Fig 1:**
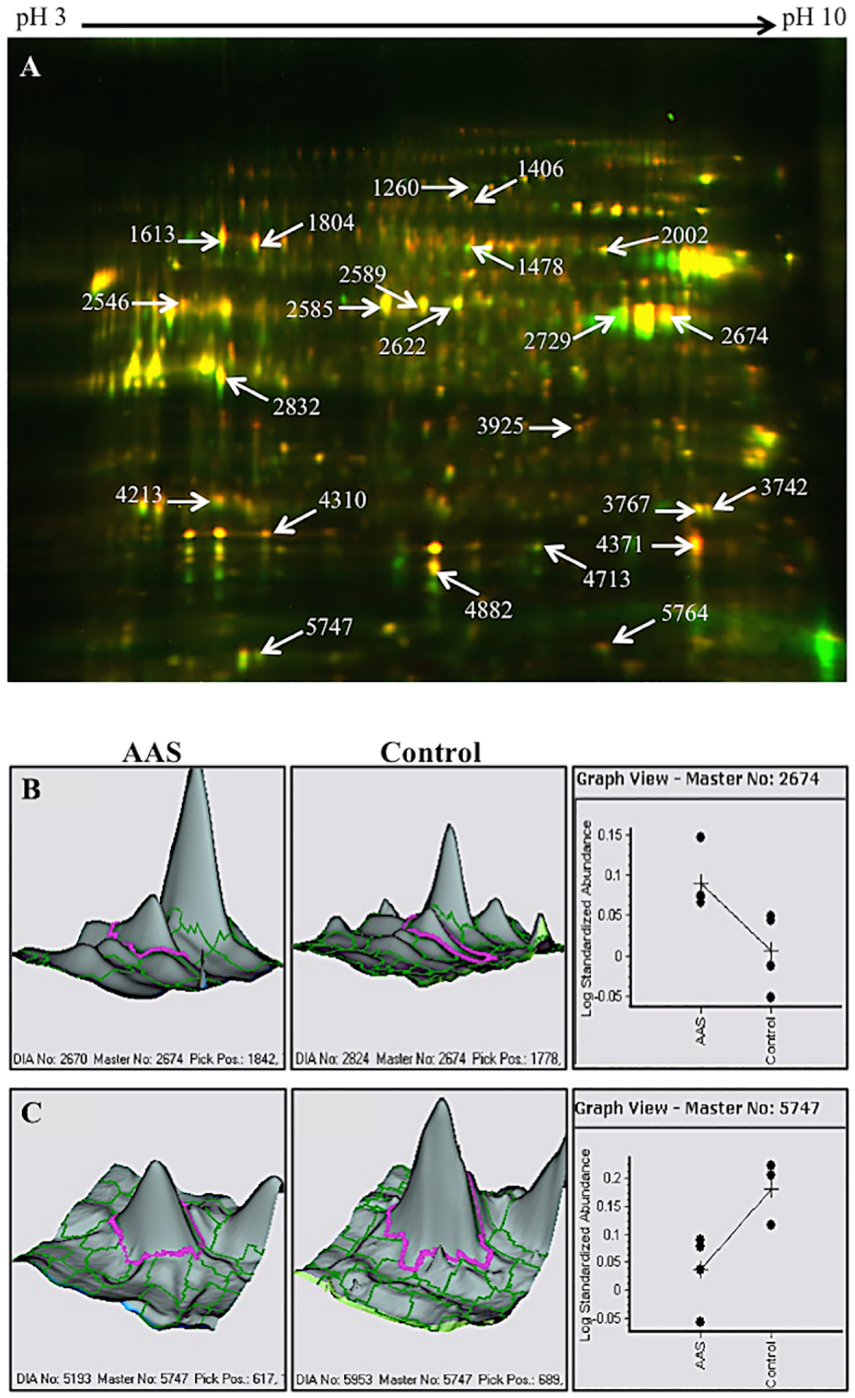
Representative images of 2D-DIGE analytical gel and 3D view of protein spots in vehicle and AAS-treated GT1-7 cells. **(A)** Twenty-three (23) protein spots were identified as differentially expressed in GT1-7 cells that were exposed to the AAS, 17α-meT (1μM). Protein samples were labeled with Cy5 (red), whereas those from the internal standard were labeled with Cy3 (green). After merging, protein spots exhibiting no changes appear yellow in DIGE images, underexpressed proteins appear in green, and overexpressed proteins are in red. White numbers and arrows correspond to the identified proteins in Tables 1 and 2. (**B-C)** Panels depict the protein spots’ abundance of **(B)** overexpressed and **(C)** underexpressed protein spots analyzed by DeCyder software. Purple lines encircle peaks representing protein spots from AAS-treated samples (left panels) or control vehicle (center panels). Protein abundance was calculated from normalized spot volume, standardized against the in-gel standard of each gel (right panels).

A total of 6,045 protein spots were detected automatically in the master 2D gel image, of which 84 protein spots were differentially expressed at a p value <0.05. From those 84 spots, only 23 were distinguishable on the preparative gel and picked for protein identification by LC-MS/MS. For easy visualization, white arrows in Fig 1A show those 23 spots from a representative analytical gel. After DeCyder analysis, spot peaks were generated. Fig 1B and 1C show examples of overexpressed (spot 2674) and underexpressed (spot 5747) protein-containing spots, respectively, during vehicle or AAS treatment.

**Fig 2:**
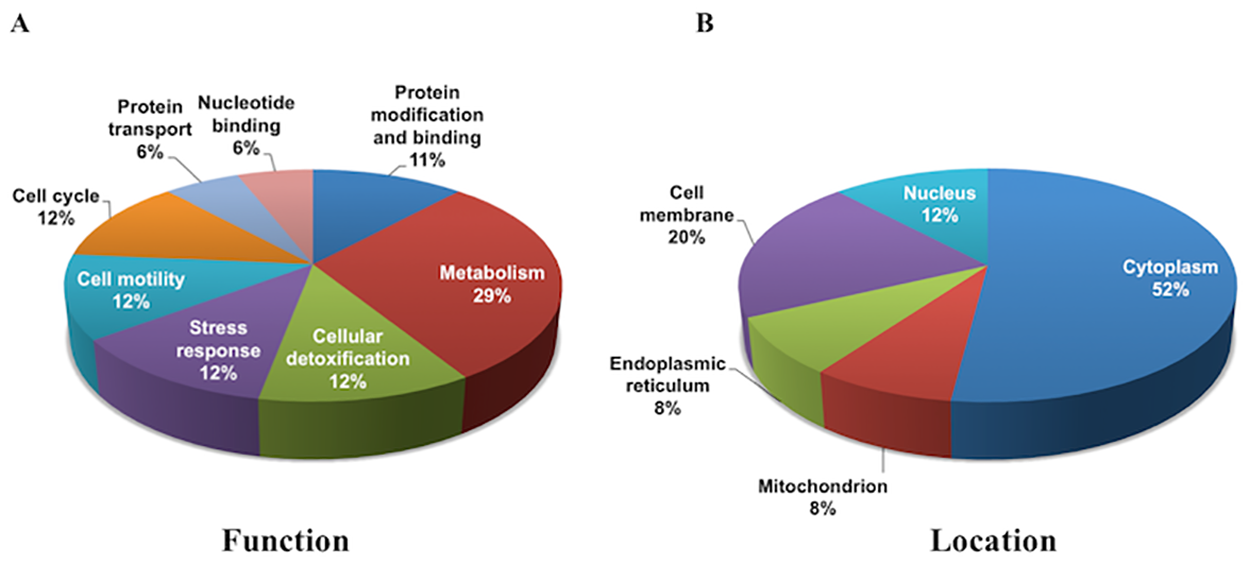
Cellular functions and locations of differentially expressed proteins in GT1-7 cells after AAS exposure. **(A)** A total of 8 biological processes with their respective cellular locations **(B)** were categorized from 17 different proteins identified by mass spectrometry. Data were obtained from Uniprot database (http://www.uniprot.org) and shown as percentages from the total number of proteins.

Tables 1 and 2 show overexpressed and underexpressed protein spots and their correspondent proteins that were differentially regulated after treatment with AAS. From those 23 recognized spots, 17 different proteins were identified. Two to four proteins were detected per protein spot collected. Top protein hits with the best Mascot scores were selected for validation as having major contribution to DIGE abundance data. UniProt database (www.uniprot.org) revealed that differentially expressed proteins after AAS treatment are related to cellular metabolism, cell cycle, cell motility, stress response, drug detoxification, as well as protein and nucleotide processing or binding (Fig 2A). Subcellular location and biological function/process analysis revealed that the majority of the identified proteins were localized in the cytoplasm, cell membrane and nucleus (Fig 2B).

**Table 1:** Overexpressed proteins in GT1-7 cells after AAS exposure.

Spot	^a^Av. Ratio	P-Value	Gene	Description/Name	Accession no.	^b^MW kDa	Peptides	Location	Function
4882	3.45	0.026	PPIA	Peptidyl-prolyl cis-trans isomerase A	P17742	18	9	Cytoplasm	Protein modification and binding
2832	1.88	0.02	SUCB2	Succinyl-CoA ligase [GDP-forming] subunit beta, mitochondrial	Q9Z2I8	47	4	Mitochondrion	Metabolism (Krebs cycle)
3742	1.51	0.043	GSTM1	Glutathione S-transferase Mu 1	P10649	26	3	Cytoplasm	Cellular detoxification
3742	1.51	0.043	GSTM2	Glutathione S-transferase Mu 2	P15626	26	7	Cytoplasm	Cellular detoxification
2729 2674	1.45 1.21	0.045 0.035	G3P (GAPDH)	Glyceraldehyde-3-phosphate dehydrogenase	P16858	36	10, 12	Cytoplasm	Metabolism (Glycolysis)
3925 3767	1.42 1.26	0.047 0.015	PGAM1	Phosphoglycerate mutase 1	Q9DBJ1	29	8, 7	Cytoplasm	Metabolism (carbohydrate metabolism)
2002	1.42	0.045	STIP1	Stress-induced-phosphoprotein 1	Q60864	63	20	Cytoplasm/Nucleus	Stress response
2546	1.41	0.023	PDIA6 (ERP)	Protein disulfide-isomerase A6	Q922R8	48	11	Endoplasmic reticulum/cell membrane	Protein modification and binding
1613 1804	1.37 1.23	0.014 0.047	HSP90B	Heat shock protein HSP 90-beta	P11499	83	26, 18	Cytoplasm	Stress response
1406	1.32	0.049	FLNA	Filamin-A	Q8BTM8	280	16	Cytoplasm	Cell motility
4371	1.32	0.031	COF1 (CFL)	Cofilin-1	P18760	18	5	Cytoplasm/cell membrane/nucleus	Cell cycle/cell motility
2585 2589 2622	1.27 1.18 1.18	0.013 0.005 0.009	ENOA (ENO1)	Alpha-enolase	P17182	47	14, 21, 23	Cytoplasm/cell membrane	Metabolism (carbohydrate metabolism)

^a^Average (Av) ratio represents the mean from four (4) different gels for both AAS-treated and control GT1-7 cells.

^b^Molecular weight

**Table 2:** Underexpressed proteins in GT1-7 cells after AAS exposure.

Spot	^a^Av. Ratio	P-Value	Gene	Description/Name	Accession no.	^b^MW kDa	Peptides	Location	Function
5747	-1.39	0.031	ERH	Enhancer of rudimentary homolog	P84089	12	5	Cell membrane	Cell cycle/RNA binding
1478	-1.50	0.0014	FLNA	Filamin-A	Q8BTM8	280	11	Cytoplasm	Cell motility
4713	-1.56	0.023	PPIA	Peptidyl-prolyl cis-trans isomerase A	P17742	18	4	Cytoplasm	Protein modification and binding
1260	-1.65	0.03	VINC (VCL)	Vinculin	Q64727	117	21	Cytoplasm/cell membrane	Cell motility
5764	-2.07	0.031	CX6A1	Cytochrome c oxidase subunit 6A1, mitochondrial	P43024	12	4	Mitochondrion	Metabolism/electron transport change
4213	-2.28	0.0021	VPS28	Vacuolar protein sorting-associated protein 28 homolog	Q9D1C8	25	3	Cell membrane/ Cytoplasm	Protein transport
4310	-2.32	0.039	PEBP1	Phosphatidylethanolamine-binding protein 1	P70296	21	5	Cytoplasm	Nucleotide binding/Protease inhibitor

^a^Average (Av) ratio represents the mean from four (4) different gels for both AAS-treated and control GT1-7 cells.

^b^Molecular weight

Overexpressed proteins in the category of glucose metabolism were: succinyl-CoA ligase [GDP-forming] subunit beta, mitochondrial (SUCB2), glyceraldehyde-3-phosphate dehydrogenase (GAPDH/G3P), phosphoglycerate mutase 1 (PGAM1), and alpha-enolase (ENOA). In this same category, cytochrome c oxidase subunit 6A1, mitochondrial (CX6A1) was underexpressed. In the category of cell motility, cofilin-1 (COF1) and vinculin (VCL) were found overexpressed and underexpressed, respectively. In proteins associated to cell cycle regulation, enhancer of rudimentary homolog (ERH) was underexpressed, while COF1 was also associated with this category. Other overexpressed proteins for the cellular stress response, were stress-induced-phosphoprotein 1 (STIP1) and heat shock protein HSP90-beta (HSP90B). Similar overexpression patterns were obtained for glutathione S-transferase Mu 1 & 2 (GSTM1 & 2), two key proteins associated with detoxification processes. On the other hand, overexpression of disulfide-isomerase A6 (PDIA6), a protein related to protein modification and binding, was also observed. In the category of transport-related proteins, vacuolar protein sorting-associated protein 28 homolog (VPS28) was found underexpressed. Finally, ERH, a protein linked to RNA binding and cell cycle, and phosphatidylethanolamine-binding protein 1 (PEBP1), a regulatory protein of nucleotide binding, were found underexpressed. Six proteins, FLNA, HSP90B, ENOA, G3P, PGAM and PPIA were represented in multiple spots and were also emphasized in Tables 1 and 2. From the 23 identified proteins, only two of them were found both overexpressed and underexpressed in different spots. These proteins were filamin (FLNA) and peptidyl-prolyl cis-trans isomerase A (PPIA), in the categories of cell motility and protein modification and binding, respectively.

### Pathway analysis

**Fig 3:**
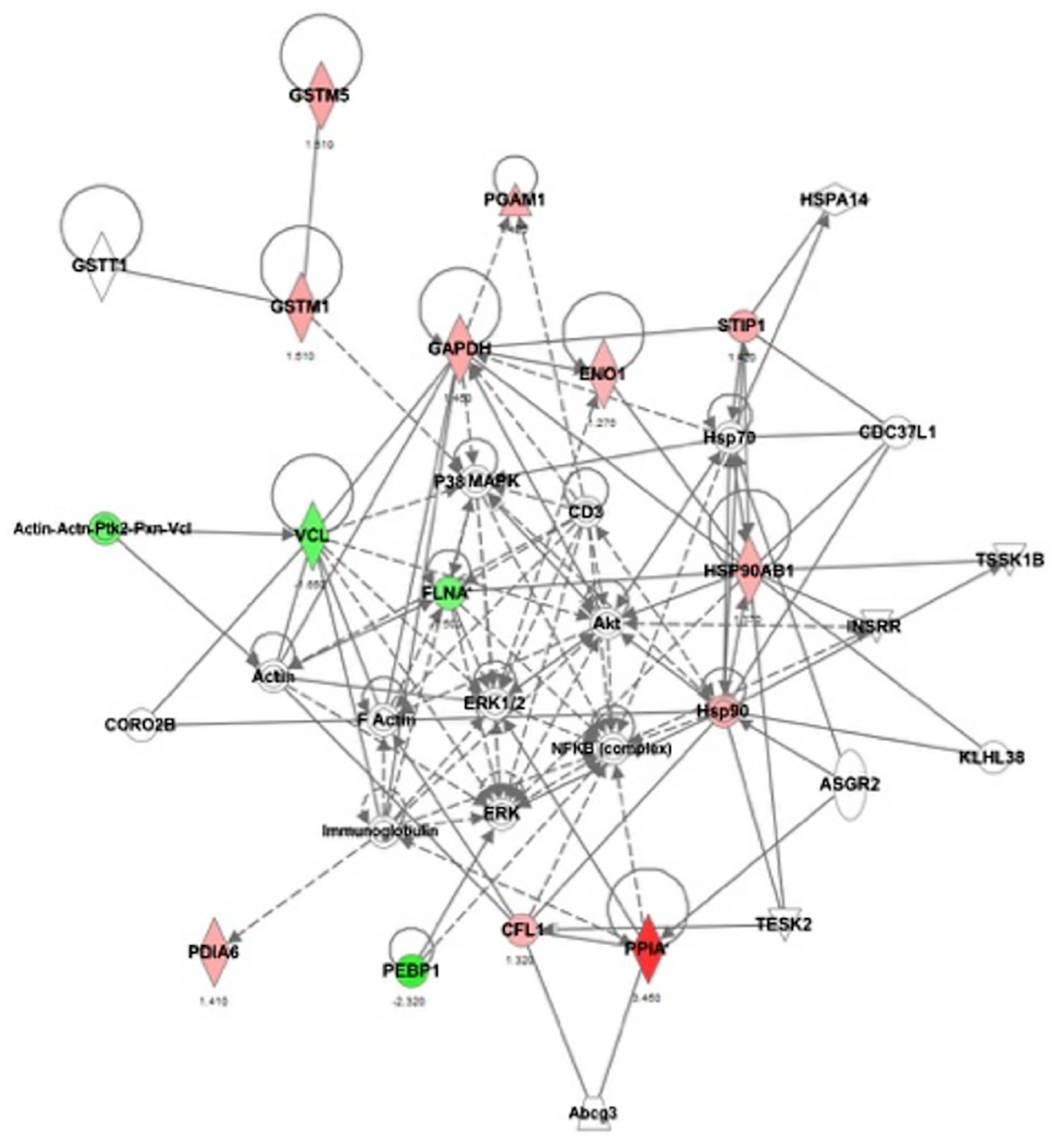
Network of proteins modulated by AAS in GT1-7 cells. Ingenuity Pathway Analysis (IPA) identified a major protein network associated with drug metabolism, glutathione depletion, protein synthesis, immunological disease and endocrine function. Red and green symbols indicate overexpressed and underexpressed proteins, respectively. GSTM2 is represented as GSTM5 in the IPA knowledge base. Direct interactions are represented as solid lines, whereas indirect interactions appear as dotted lines.

Using the Ingenuity^**®**^ Pathway Analysis software (IPA), we provided insights into AAS-induced protein expression changes in the GT1-7 cell line. These networks are ranked by scores, and are based on the number of focus proteins and the size of the network. Scores of 10 or higher (negative log of the P value) have high confidence, avoiding random effects [36]. The pathway analysis identified only one network with a high score of 35, and it was associated to drug metabolism, glutathione depletion, protein synthesis, immunological disease, and endocrine system development and function. The network incorporated 13 focus proteins out of the 17 identified proteins modulated by 17α-meT (Fig 3). The cannonical signaling pathway showed the highest significance and number of focus proteins for glycolysis (3 proteins), gluconeogenesis (3 proteins), aryl hydrocarbon receptor (AHR signaling; 3 proteins), glutathione mediated detoxification (2 proteins) and NRF2-meditaited oxidative stress response (3 proteins). Other signaling pathways associated with AAS-induced protein modulation are shown in Table 3.

**Table 3:** Canonical pathways modulated in GT1-7 cells after AAS exposure.

Pathway	-log (p-value)	Ratio	Molecules
Glycolysis	5.94E00	1.2E-01	ENO1, GAPDH, PGAM1
Gluconeogenesis	5.94E00	1.2E-01	ENO1, GAPDH, PGAM1
Aryl hydrocarbon receptor signaling	3.68E00	2.14E-02	GSTM1, GSTM2, HSP90B
Glutathione-mediated detoxification	3.54E00	6.67E-02	GSTM1, GSTM2
NRF2-mediated oxidative stress receptor	3.36E00	1.67E-02	GSTM1, GSTM2, STIP1
Actin cytoskeleton signaling	3.12E00	1.38E-02	CFL1, FLNA, VCL
Xenobiotic metabolism signaling	2.84E00	1.11E-02	GSTM1, GSTM2, HSP90B
NADH repair	2.6E00	3.33E-01	GAPDH
4-aminobutyrate degradation I	2.6E00	3.33E-01	SUCLG2 (SUCB2)
Rapoport-Luebering glycolytic shunt	2.47E00	2.5E-01	PGAM1
Glutamate degradation III (via 4-aminobutyrate)	2.37E00	2E-01	SUCLG2 (SUCB2)
ILK signaling	1.98E00	1.08E-02	CFL1, FLNA
LPS/IL-1 meditated inhibition of RXR function	1.84E00	9.13E-03	GSTM1, GSTM2
Maturity onset diabetes of young (MODY) signaling	1.73E00	4.55E-02	GAPDH
Mechanism of viral exit from host cells	1.47E00	2.44E-02	VPS28
Ephrin A signaling	1.4E00	2.08E-02	CFL1
Semaphorin signaling in neurons	1.36E00	1.89E-02	CFL1
Regulation of cellular mechanics by calpain protease	1.33E00	1.75E-02	VCL

Dataset generated by Ingenuity Pathway Analysis

### Confirmation of proteomic changes by western blotting

**Fig 4:**
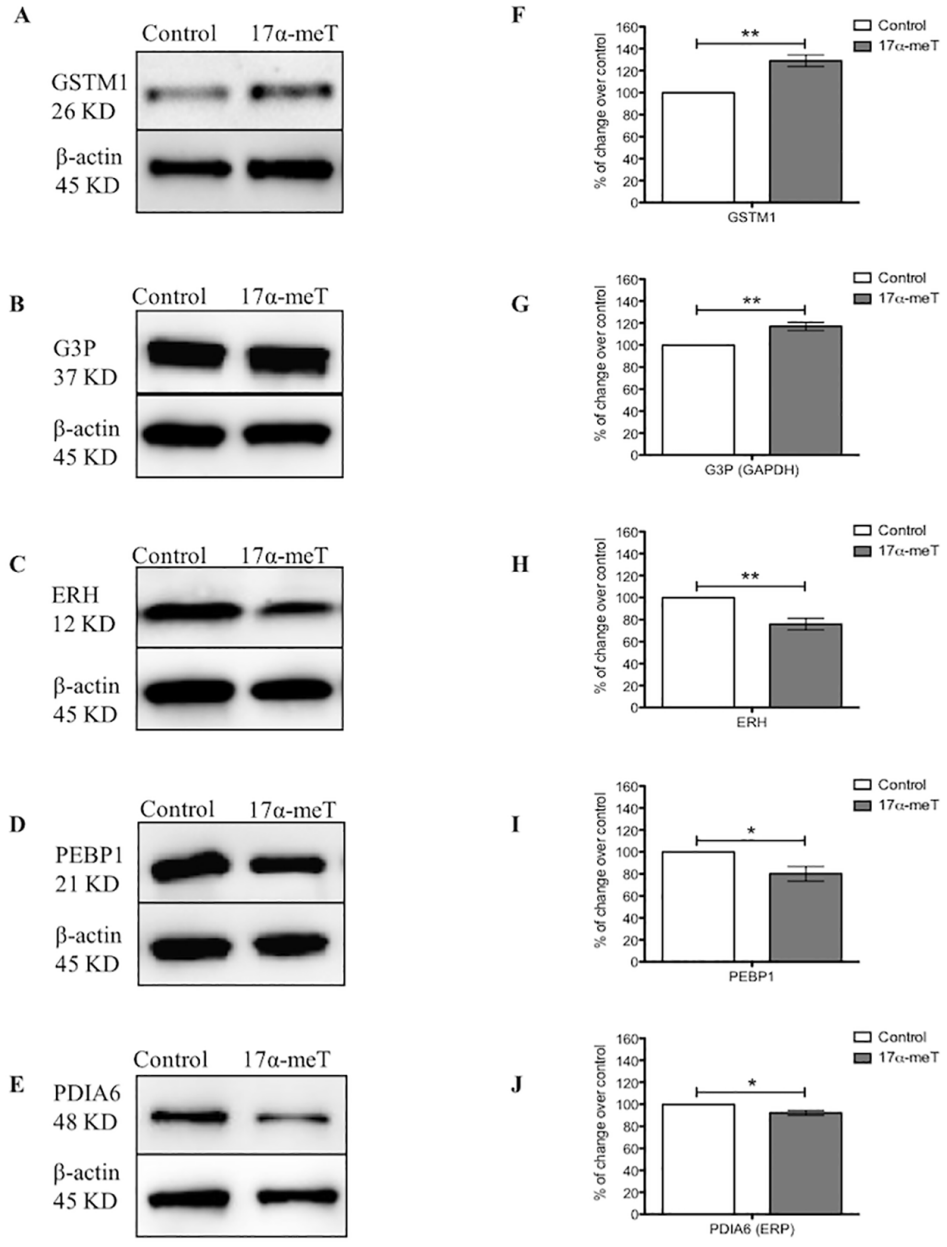
Validation of differentially expressed proteins in GT1-7 cells after AAS exposure. **(A-E)** Representative Western blots of identified proteins from protein extracts of AAS or vehicle-treated cells. **(F-J)** Densitometry analysis of each protein normalized to β-actin represents the relative protein expression values for **(A)** Glutathione S-transferase Mu 1 (GSTM1), **(B)** Glyceraldehyde 3-phosphate dehydrogenase (G3P/GAPDH), **(C)** Enhancer of rudimentary homolog (ERH), **(D)** Phosphatidylethanolamine-binding protein 1 (PEBP1), **(E)** Protein disulfide-isomerase A6/Endoplasmic Reticulum Protein (ERP/PDIA6). Error bars represent standard error of the mean. *p≤0.05, **p≤0.01, unpaired t-test. n = three replicates of three independent experiments for each group.

**Fig 5:**
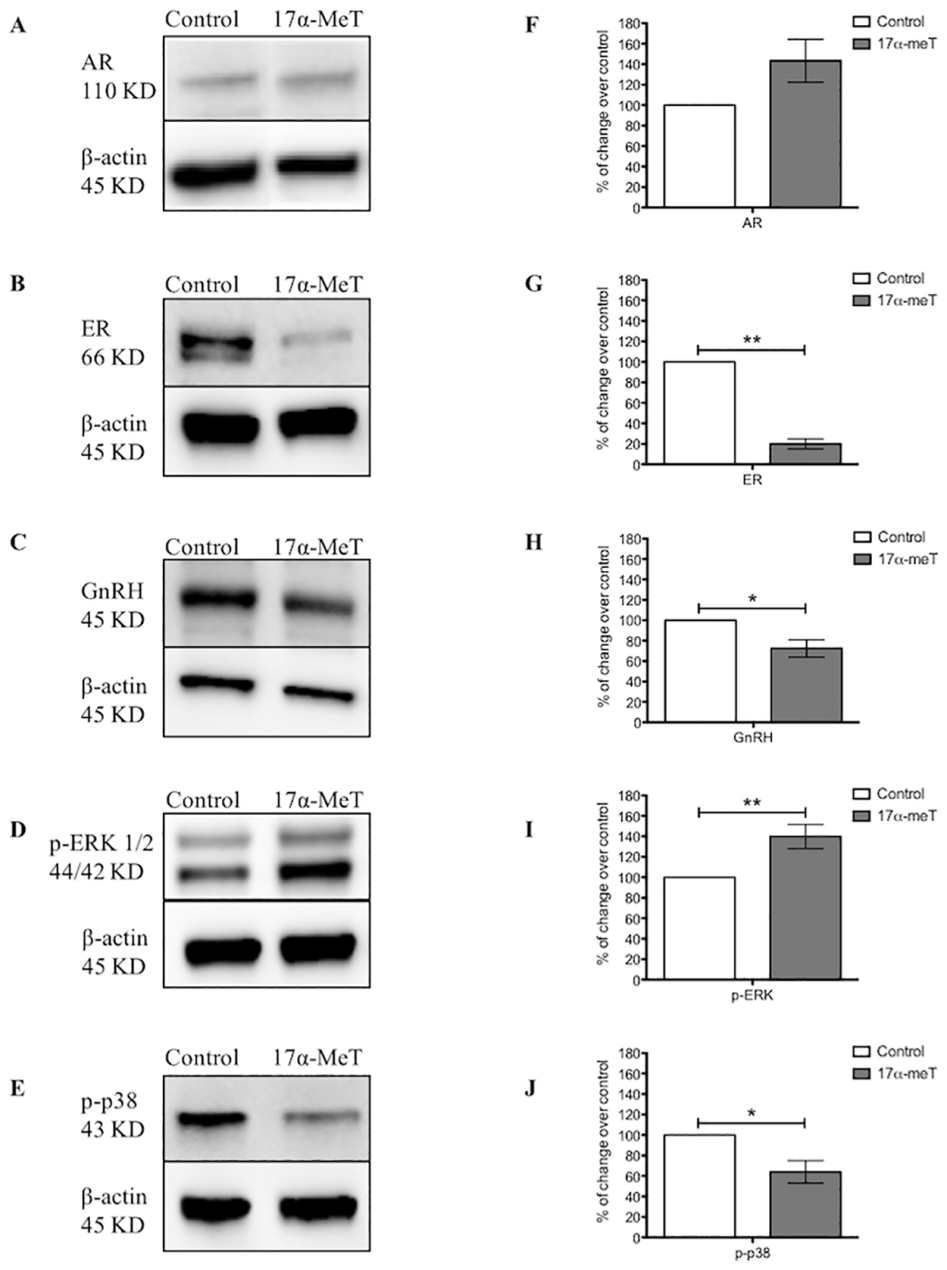
Protein expression of steroids receptors and signaling pathway proteins in GT1-7 cells after AAS exposure. **(A-E)** Representative Western blots of AR, ER, GnRH, p-ERK and p-p38 from protein extracts of AAS or vehicle-treated cells. **(F-J)** Densitometry analysis of each protein normalized to β-actin represents the relative protein expression values for **(A)** Androgen receptor (AR), **(B)** Estrogen receptor (ER), **(C)** GnRH, **(D)** Phospho-p44/42 MAPK (Erk1/2; Thr202/Tyr204), and **(E)** Phospho-p38 MAPK (p-p38), Error bars represent standard error of the mean. *p≤0.05, **p≤0.01, unpaired t-test. n = three replicates of three (AR and ER) or four (GnRH, p-ERK and p-p38) independent experiments for each group.

In order to validate proteins that were differentially expressed in GT1-7 cells by AAS, we performed western blotting on cell lysates for several proteins in different categories. We found that all proteins tested by Western blots were expressed in GT1-7 cells. These were: GSTM1, GAPDH/G3P, ERH, PEBP1, and PDIA6. Thereafter, we performed Western blots to GT1-7 cell lysates in order to determine the expression of these proteins after steroid treatment. We confirmed that similar to the proteomic results, treatment with 17α-meT increased the expression of GSTM1 and G3P (Fig 4A and 4B) and decreased expression of ERH and PEBP1 (Fig 4C and 4D). PDIA6 was observed to be underexpressed in Western blots, while 2D-DIGE revealed overexpression. Finally, in order to evaluate modulatory effects of AAS in classical endocrine substrates, we interrogated the expression of AR, ER and GnRH, as well as proteins associated to signaling pathway proteins (p-ERK, ERK, p-p38 and AKT). Densitometry analyses showed that 17α-meT did not significantly change AR (Fig 5A), whereas ER (Fig 5B) and intracellular GnRH (Fig 5C) were decreased. Regarding the expression of signaling proteins, AAS exposure increased p-ERK expression (Fig 5D), while p-p38 (Fig 5E) was downregulated. On the other hand, expression of the non-phosphorylated forms, ERK and AKT did not change, as observed by the % of change over control (data not shown).

## Discussion

The current study identified changes in the proteome of the hypothalamic GT1-7 cells in response to the synthetic androgen, 17α-meT. Since physiological levels of androgens have been determined as 0.01 μM [31,32], the dose regimen used in our study is considered a chronic supraphysiological (100-fold) exposure (1 μM for 48 h) [27]. In fact, this steroid dosage reflects the regimen used by professional and amateur athletes who currently use 10 to 100-fold doses of AAS to improve their athletic performance [38–40]. In humans, although basal testosterone levels of cerebrospinal fluid (CSF) ranged between 0.0001–0.001 μM [41,42], systemic exposure to high levels of methyltestosterone during 6 days (40–240 mg/day) led to concentrations in the CSF that ranged between 0.065–0.9 μM [43]. This dose is similar to the dose tested in our *in vitro* study. These results suggest that cell populations in the brain of AAS abusers can be under the direct influence of supraphysiological concentrations of the drug, and that anabolic steroids have the potential to cause negative consequences at the molecular, physiological and/or behavioral levels.

Although androgen doses between 1 to 10 μM initiated apoptosis in a neuroblastoma cell line [33], in our study, 1 μM of AAS did not affect the viability of GT1-7 neurons, nor did they affect caspase activity. In this regard, two other cell lines have been differentially affected by androgens. Specifically, dopaminergic N27 neurons showed androgen-induced apoptosis, whereas GT1-7 cells were not affected [44]. Interestingly, others have shown that exposure to methandrostenelone (1 μM), an AAS that belongs to the same category as 17α-meT, reported cell viability that ranged between 80–95% [27]. To further argument against possible treatment-induced toxic effects, we showed that physiological levels of dihydrotestosterone (DHT, 0.1 μM) overexpressed pERK (131.86% over control; p = 0.026, unpaired t-test), similar to what we observed by 17α-meT (1 μM). In accordance with our data, another study suggested that androgen-induced-neuroprotection might be achieved through overexpression/activation of ERK signaling [45]. Therefore, given that, in our study, the integrity of the cells was not compromised by a supraphysiological dose of androgens, we showed that, under these conditions, GT1-7 cells revealed protein changes in the categories of cellular metabolism, drug detoxification, stress response, cell cycle and motility, as well as in nucleotide binding and protein modification and transport.

### Metabolism

In the metabolism category, the most significantly overexpressed proteins belong to the glycolysis and gluconeogenesis (5.94E00) pathways. In general, chronic exposure to androgens is associated with insulin resistance, glucose intolerance, low glucose disposal rate and diabetes [46]. In neurons, increments in glycolytic enzymes are related to ion pumps recruitment, stimulation of glial glycolysis and glucose uptake [12,47]. In the rat epididymis, exposure to testosterone and DHT (1 mg/kg b.w.) increased the activity of GAPDH and PGAM [48]. Beside changes in metabolism, these proteins have been associated with other biological processes. For example, increased GAPDH expression has been linked to proliferation, apoptosis, cytoskeleton organization and synaptic remodeling [49]. Similarly, increments in ENOA and PGAM1 have been associated with tumor proliferation, cell migration, and apoptosis [50,51]. On the other hand, SUCB2, an enzyme of the tricarboxylic acid cycle (TCA) was found overexpressed, and associated by the canonical analysis with 4-aminobutyrate degradation I (2.6E00) and glutamate degradation III (via 4-aminobutyrate; 2.37E00). In this regard, others have shown that gene overexpression of TCA enzymes is related to high steroidogenic activity [52].

### Drug detoxification and stress response

Regarding the possibility of 17α-meT inducing noxiousness and processes associating drug detoxification and stress response, we observed overexpression of two glutathione-mediated detoxification proteins (3.54E00), GSTM1 and GSTM2. GSTM proteins are steroid binding proteins characterized by their properties to bind testosterone and estradiol [53]. Upregulation of these antioxidant proteins has been related to drug insult, and detoxification of xenobiotics and oxidative products [54]. Interestingly, GSTM1 was upregulated by supraphysiological doses of DHT in peripheral human lymphocytes [26]. As well, corticosterone treatment in mice increased GSTM1 expression in steroid sensitive brain regions, such as the hypothalamus, hippocampus and cortex [36]. We also observed a modulation in the NRF2-mediated oxidative stress receptor pathway (3.36E00), associated with overexpression of the antioxidant and stress response proteins, GSTM and STIP1. This pathway has also been associated with a decreased expression of PEBP1, a Raf kinase inhibitory protein (RKIP). Therefore, it is not surprising that, in our study, PEBP1 was found to be decreased by 17α-meT.

The heat shock protein, HSP90B, was found overexpressed and associated with aryl hydrocarbon receptor (AHR; 3.68E00) and xenobiotic metabolism (2.84E00) pathways. HSP90B commonly acts as a chaperone molecule facilitating the proper folding of proteins in ATP-dependent reactions. This protein is also induced upon stress and xenobiotic exposure [55]. HSP90B also forms associations with AHR’s and glucocorticoid receptors, which bind foreign compounds, including steroids [56]. Furthermore, AHR’s may interact with a number of xenobiotic-binding proteins such as steroid receptors [56] and cytochrome P450, which also metabolize androgens [57], estrogens [58] and AAS [59].

### Cell motility and cell cycle

Proteins related to cell motility and cytoskeleton dynamics were modulated after exposure to 17α-meT. This was represented by changes in VCL, FLNA, and CFL1 (3.12E00). VCL, a structural protein regulating cell-cell adhesion [60], showed decreased expression. Accordingly, the progesterone metabolite, 5a-pregnane-3, 20-dione, decreases both VCL expression and cell adhesion, suggesting proliferative and metastatic inductions by an endogenous steroid [61]. Also, activation of androgen receptors correlates with VCL inhibition, restoring the migration potential of cancerous cells [62].

The scaffolding protein, FLNA, is linked to steroid-induced cell motility [63]. Although we observed this protein to be overexpressed and underexpressed in two different spots, which could suggest posttranslational modifications (PTM’s) and isoform regulation, FLNA has been associated to the formation of complexes with androgen receptors in response to steroids. This type of regulation has been documented in processes of neurogenesis, cell migration and invasiveness [64]. Additionally, FLNA facilitates nuclear translocation of the androgen receptor to modulate gene expression [65].

CFL1, a regulatory protein of mitosis and cell migration processes (www.uniprot.com), was found overexpressed after 17α-meT exposure. This protein acts through depolymerization of actin filaments by regulating the cytoskeleton dynamics and cell morphology [66]. Several reports indicate that steroids mediate the CFL1 phosphorylation pathway, inducing actin filament elongation [67]. Specifically, estrogen stimulates LIM kinase (LIMK)-dependent phosphorylation of CFL1, which promotes filopodial extensions and spine synapse growth. Increments in LIMK activity and CFL1 phosphorylation also correlate with cell proliferation [68] and migration [69].

ERH, another cell cycle protein, showed decreased expression in response to 17α- meT. Although the function of the protein is not well known, it has been associated to cell cycle, RNA binding/splicing and cancer processes [70]. ERH underexpression produces defects in chromosomal congression during mitosis [70,71]. Specifically, ERH depletion results in the loss of the mitotic motor protein, CENP-E, of the kinetochore, increasing mitosis duration. This depletion downregulates other genes involved in DNA replication and repair, suggesting that ERH is essential for chromosomal segregation and cell cycle progression [70,71]. Other experiments suggest that ERH underexpression may constrain tumor aggressiveness, given that ERH knockdown reduces tumor cell viability through KRAS oncogene-dependent pathways [70,72].

### Protein modification and binding

PEBP1 (RKIP), a protease inhibitor, and also a protein related to nucleotide binding [73], was found underexpressed after exposure to 17α-meT. PEBP1 has been associated with suppression of metastasis [73]. Specifically, reduced expression of PEBP1 is highly associated to metastatic cancers, such as prostate metaplasia. Therefore, although a physiological dose of DHT (10 nM) increased PEBP1 expression in prostate epithelial cells [74], suggesting androgen anticancer properties, our results suggest that supraphysiological doses of steroids might induce detrimental tumorigenic and degenerative events.

PDIA6 and PPIA, two isomerases involved in protein modification and folding, were modulated in response to AAS treatment. Increased PDIA6 is associated to unfolded proteins response (UPR), a biological process that accumulates misfolded proteins in the endoplasmic reticulum, as a result of stress signals [75]. Although, 2D-DIGE analyses showed an overexpression of the spot containing PDIA6, a decreased was observed by western blotting. Discrepancies in both techniques have been previously reported [76,77], since 2D-DIGE detects PTM’s and protein isoforms, whereas western blotting detects the overall protein signal [78]. Nonetheless, using 2D-DIGE, has been demonstrated that exposure to high testosterone doses overexpressed PDIA6 in rat meiotic spermatocytes [79]. Moreover, PPIA is associated with protein folding, lipid organization and stress response (www.uniprot.com). Although 17α-meT might be inducing PTMs on PPIA, as observed by over and underexpressed spots, increased expression of this protein has been related to dexamethasone [80] and estrogen [81] exposure.

### Classical steroid receptors and GnRH signaling pathways

Previous studies demonstrated that exposure to androgens decreased GnRH in GT1-7 cells [9,12]. For instance, in these cells, several studies reported that DHT and testosterone (1–10 nM) [9,10,27], as well as DHEA (10^−4^ M), decreased GnRH transcript [28,82]. In the same line of evidence, our data showed that 17α-meT decreased intracellular GnRH expression, suggesting repressive effects on FSH and LH, hormonal changes that might represent dysregulation of the endocrine system. However, we observed that 17α-meT caused a non-significant trend to increase AR expression. Similar to our results is the fact that DHT (2.4 μM) did not modulate the expression of AR in lymphocytes [26]. While modulating proteins associated with cellular proliferation, cell death and drug metabolism. Interestingly, we found that AAS exposure decreased expression of ER, a result that is consistent with previous studies showing that, in the hypothalamus, its expression is inhibited by androgens [83].

Sex steroids have been found to modulate signal transduction pathways [84]. In this regard, p-ERK has been extensively related to cell proliferation, survival and plasticity [85,86]. While some studies suggest that androgens might reduce p-ERK expression and induce apoptosis [87], other studies demonstrate that testosterone activates p-ERK expression and proliferation [88,89]. The fact that we found proteins associated to cell proliferation, such as VCL, FLNA, GAPDH and STIP1, is in accordance with the proliferative effect of p-ERK. Therefore, it is suggested that these proteins might act as upstream regulators of ERK signaling pathways. Moreover, stimulation of cytoplasmic AR by anabolic steroids undergoes non-genomic signaling cascades leading to p-ERK activation and cell proliferation [90]. Interestingly, in our study, neither the non-phosphorylated forms of ERK nor AKT changed their expression after AAS exposure. On the other hand, the stress-activated kinase p-p38 [91] was downregulated, suggesting negative feedback for the restoration of cellular homeostasis after hormonal imbalance. This is in accordance with a previous study showing that acute stimulation of myotubes with supraphysiological doses of testosterone reduced phosphorylation of p38 MAPK [92].

### Conclusion

By interrogating the proteome of the hypothalamic GT1-7 cell line as a model for studying AAS-induced regulatory processes of reproduction, we found that anabolic steroids modulate proteins associated to metabolism, cytoarchitecture, cellular homeostasis and hormonal regulation. Regarding possible direct androgen actions, it is worth mentioning that several of the identified proteins have androgen-response elements (ARE’s). Specifically, the Androgen Responsive Gene Database (http://argdb.fudan.edu.cn) [93] revealed that proteins involved in metabolism (GAPDH, PGAM1, ENOA) stress response (STIP1, HSP90B) drug detoxification (GSTM1-2), protein modification (PEBP1, PPIA, PDIA6), and signaling pathway (ERK), contain ARE’s. Although our findings provide a comprehensive molecular evaluation of how AAS might affect neurosecretory hypothalamic neurons, it is noteworthy to mention that *in vitro* studies using cell lines provide limitations when extrapolating results *in vivo*. Among the limitations in our study is the lack of complexity in the intact hypothalamic architecture, including afferent connections from distinct hypothalamic nuclei and neurons. In this scenario, it is still missing the neuronal interaction through neuropeptide/neurotransmitter signaling between GnRH neurons, feeding-related neurons and those from the suprachiasmatic nucleus. Despite these limitations, cell lines have proven themselves to be good models that are helpful in order to understand the *in vivo* complexity by providing a simpler system that is easy to maintain. This system has a homogeneous population of neurons, where controllable variables can be studied [94]. Therefore, differentially expressed proteins found in our study represent potential biomarkers of GnRH hypothalamic neurons for the detection of AAS-induced changes in reproductive function. It is then suggested that in steroid abusers, typical biological processes related to reproduction might be compromised through: i) activation of the biological machinery to reestablish cellular homeostasis after stressful and metabolic events, and/or ii) cellular proliferation and migration that might induce tumorigenic activity. Future studies will be needed to test these modulated proteins against the physiology of reproduction.

## Supporting information

S1 File Western blot membranes and antibodies information for (A) GSTM1, (B) GP3/GAPDH, (C) ERH, (D) PEBP1, (E) PDIA6/ERP. Each antibody is normalized to β-actin (right panels).
(PDF)

S2 File Western blot membranes and antibodies information for (A) AR, (B) ER, (C) GnRH, (D) p-ERK, (E) p-P38. Each antibody is normalized to β-actin (right panels).
(PDF)
