# Supplementary material for: Differential protein expression profile in the hypothalamic GT1-7 cell line after exposure to anabolic androgenic steroids
Source: PLoS One. 2017 Jul 18;12(7):e0180409. doi: 10.1371/journal.pone.0180409 (PMC5515402; doi:10.1371/journal.pone.0180409)
Supplement: S1 File — Each antibody is normalized to β-actin (right panels). (PDF) [file pone.0180409.s001.pdf]

Supporting Information

(For figure 4)

S1-A

GSTM1 (26 KD)

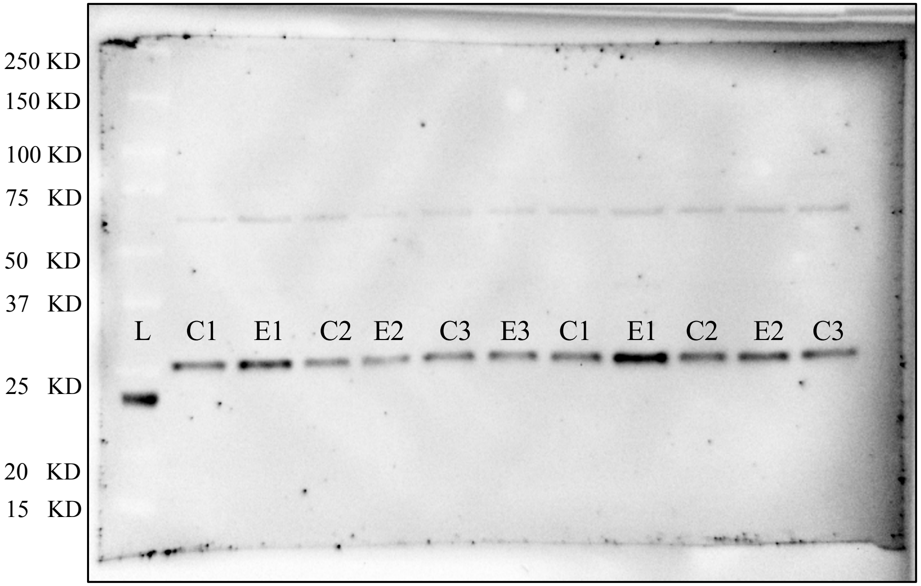

- GSTM1 (IgG)
- PA5-22278
- Polyclonal
- Company: Thermo Scientific

β-actin (45 KD)

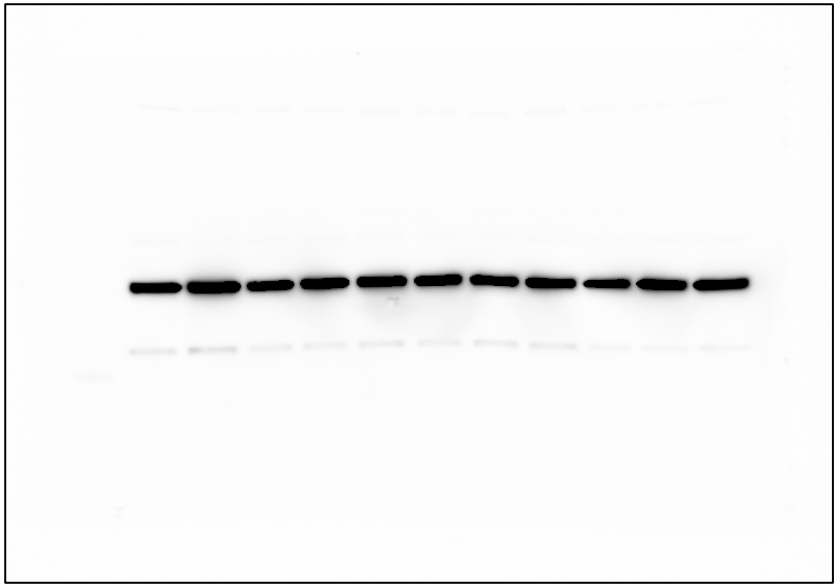

- β-Actin (13E5) Rabbit mAb (HRP Conjugate)
- #5125
- Monoclonal
- Company: Cell Signaling

L= ladder
C=control samples (vehicle)
E= experimental samples (AAS)

S1-B

GP3/GAPDH (37 KD)

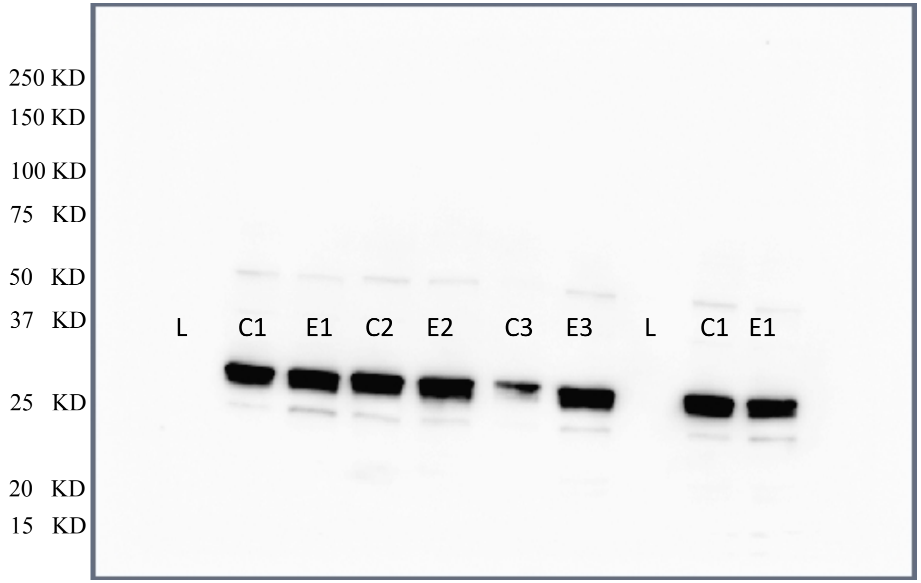

- GAPDH (D16H11)XP Rabbit mAb (HRP Conjugate)
- #8884
- Monoclonal
- Company: Cell Signaling

β-actin (45 KD)

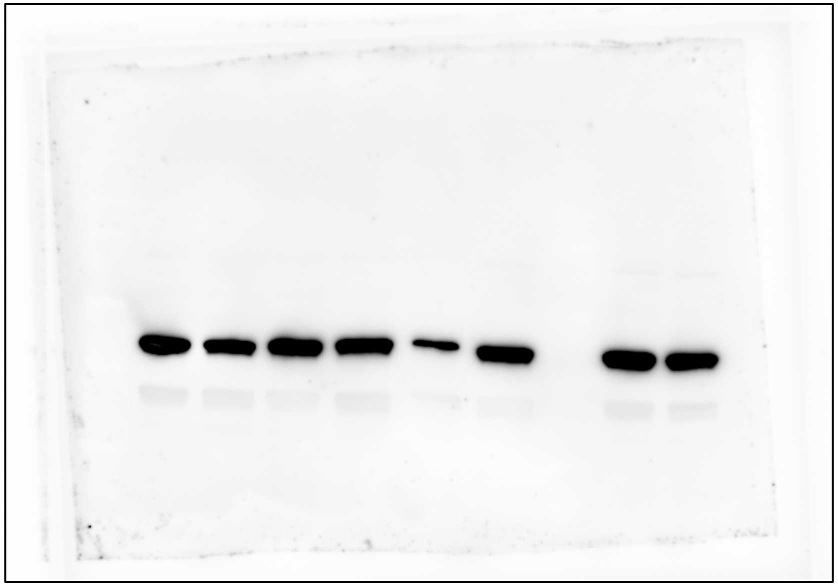

- β-Actin (13E5) Rabbit mAb (HRP Conjugate)
- #5125
- Monoclonal
- Company: Cell Signaling

L= ladder
C=control samples (vehicle)
E= experimental samples (AAS)

S1-C

ERH (12 KD)

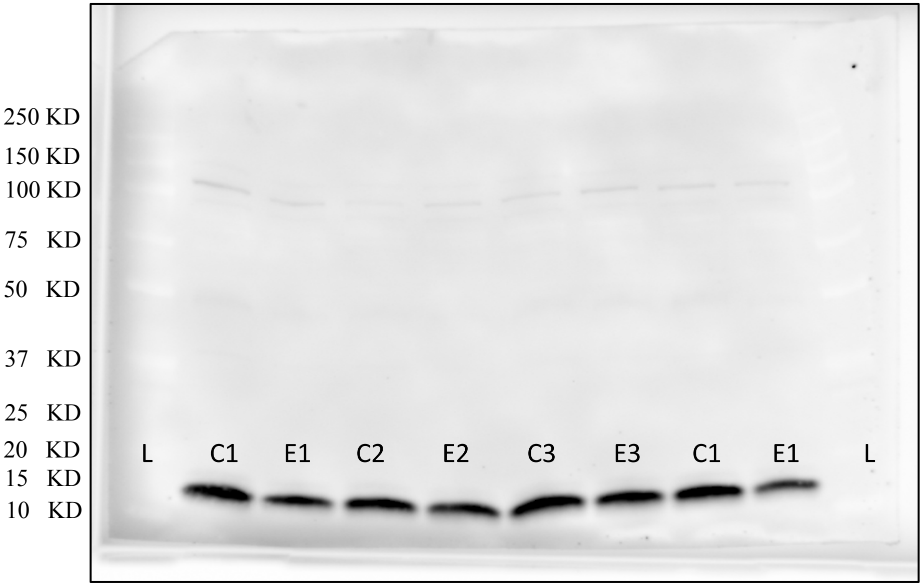

- ERH (H-8) Mouse
- Sc-373957
- Monoclonal
- Company: Santa Cruz

β-actin (45 KD)

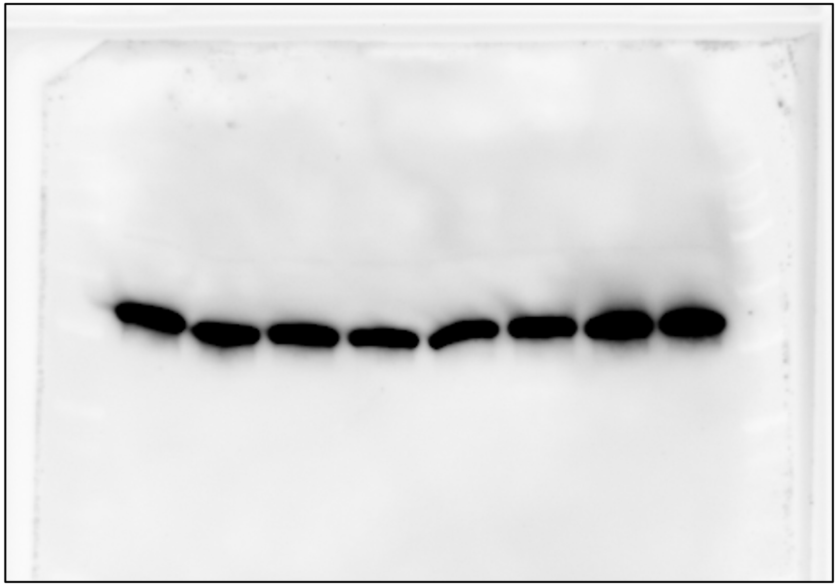

- β-Actin (13E5) Rabbit mAb (HRP Conjugate)
- #5125
- Monoclonal
- Company: Cell Signaling

L= ladder
C=control samples (vehicle)
E= experimental samples (AAS)

S1-D

PEBP1 (21 KD)

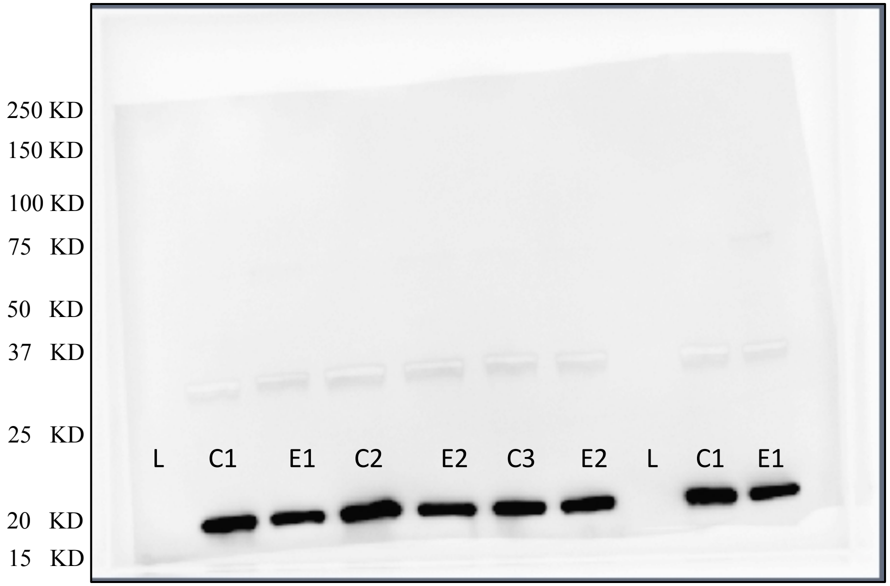

- PEBP1/RKIP (D42F3) Rabbit mAb
- #13006
- Monoclonal
- Company: Cell Signaling

β-actin (45 KD)

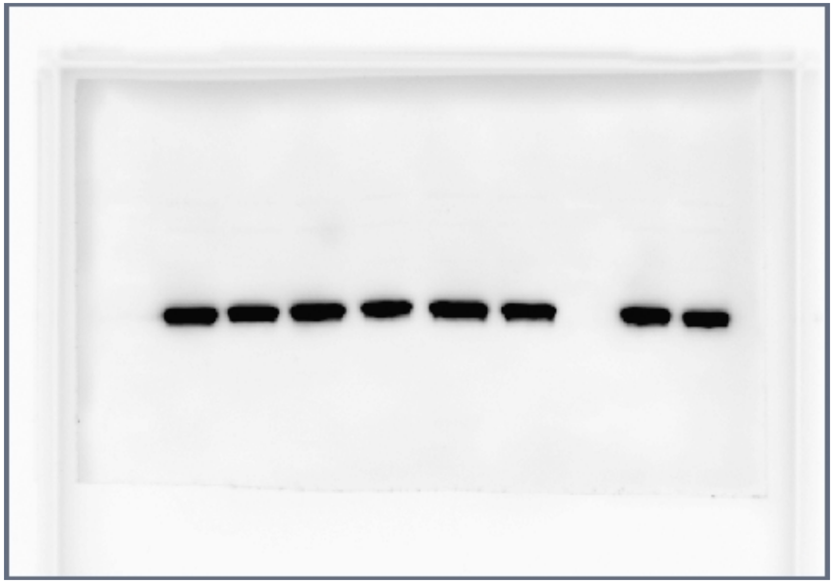

- β-Actin (13E5) Rabbit mAb (HRP Conjugate)
- #5125
- Monoclonal
- Company: Cell Signaling

L= ladder
C=control samples (vehicle)
E= experimental samples (AAS)

S1-E

PDIA6/ERP (48 KD)

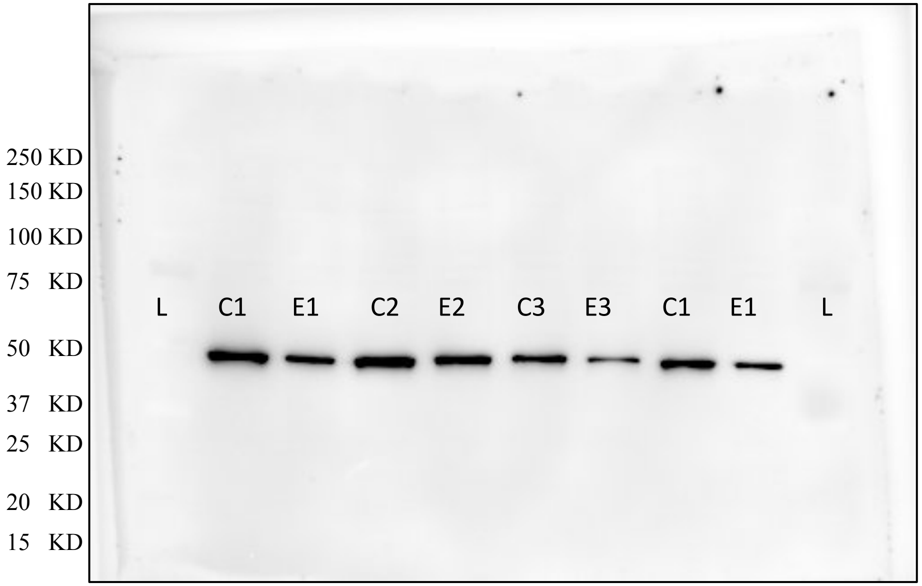

- PDIA5/ERp5 (G-5) Mouse
- Sc- 365260
- Monoclonal
- Company: Santa Cruz

β-actin (45 KD)

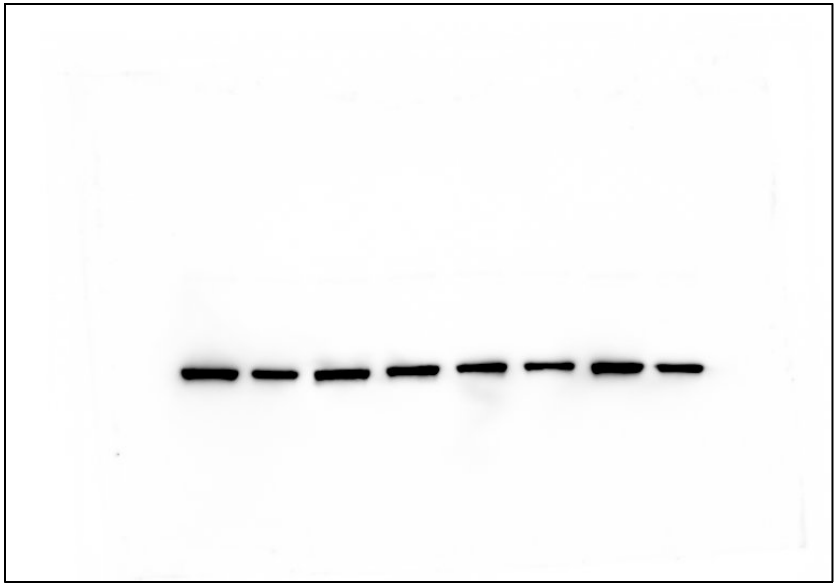

- β-Actin (13E5) Rabbit mAb (HRP Conjugate)
- #5125
- Monoclonal
- Company: Cell Signaling

L= ladder
C=control samples (vehicle)
E= experimental samples (AAS)
