# Supplementary material for: Differential protein expression profile in the hypothalamic GT1-7 cell line after exposure to anabolic androgenic steroids
Source: PLoS One. 2017 Jul 18;12(7):e0180409. doi: 10.1371/journal.pone.0180409 (PMC5515402; doi:10.1371/journal.pone.0180409)
Supplement: S2 File — Each antibody is normalized to β-actin (right panels). (PDF) [file pone.0180409.s002.pdf]

Supporting Information

(For figure 5)

S2-A

Androgen Receptor (110 KD)

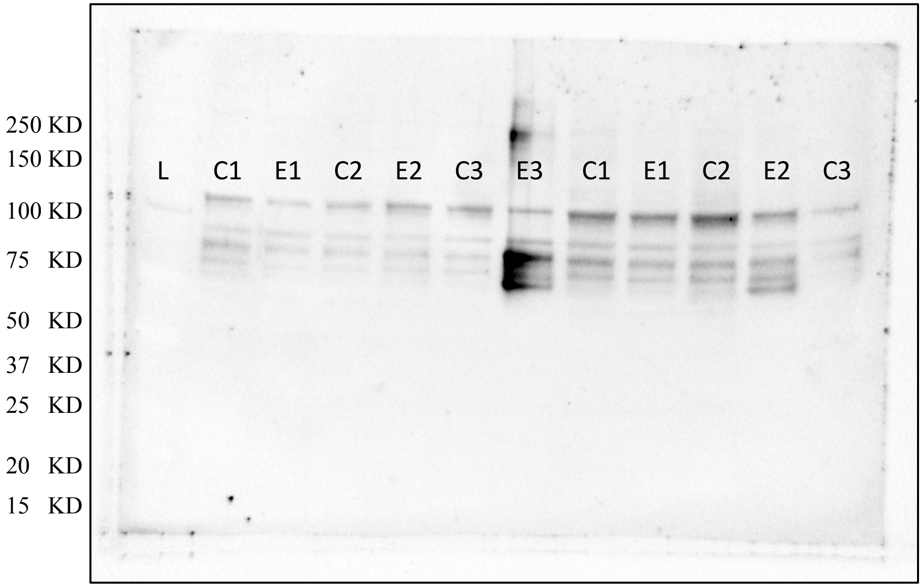

- AR (G13) Goat
- Sc- 109500
- Polyclonal
- Company: Santa Cruz

β-actin (45 KD)

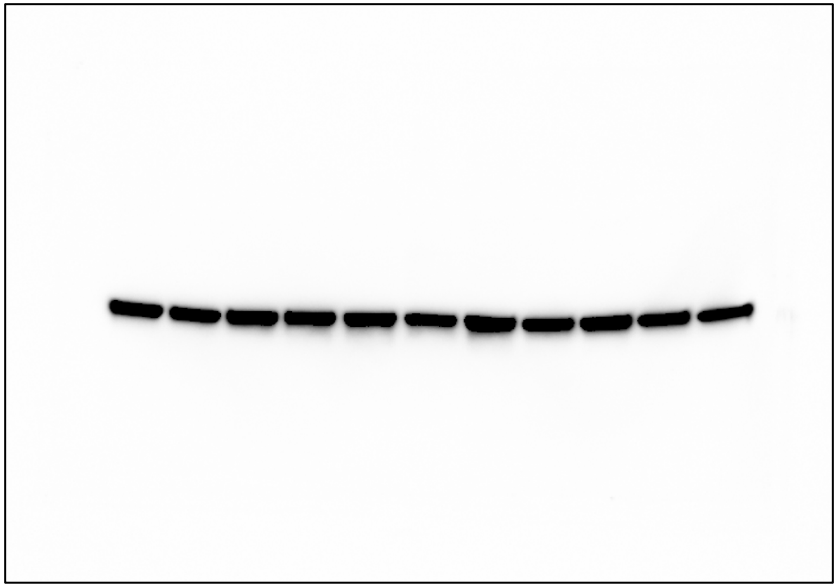

- β-Actin (13E5) Rabbit mAb (HRP Conjugate)
- #5125
- Monoclonal
- Company: Cell Signaling

L= ladder
C=control samples (vehicle)
E= experimental samples (AAS)

S2-B

Estrogen Receptor (66 KD)

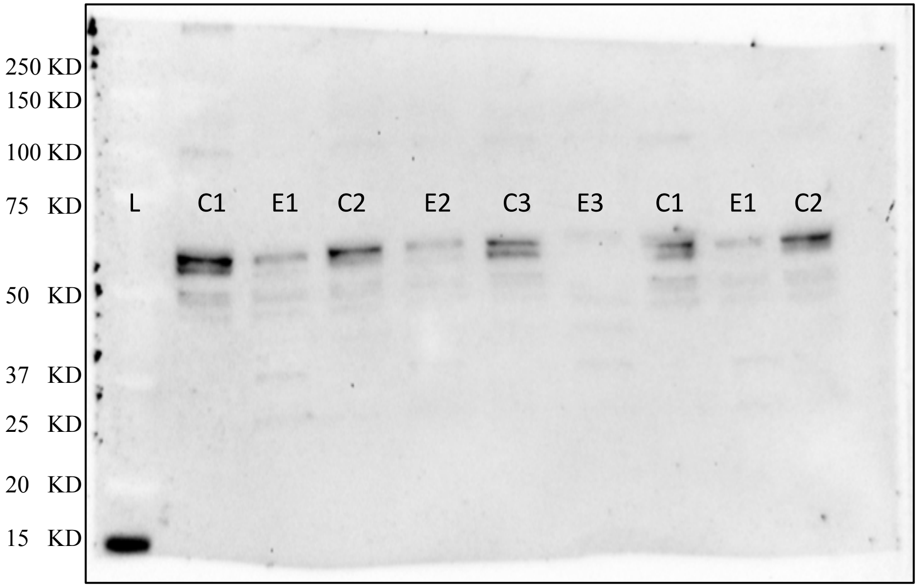

- ERα (MC-20) Rabbit
- Sc- 542
- Polyclonal
- Company: Santa Cruz

β-actin (45 KD)

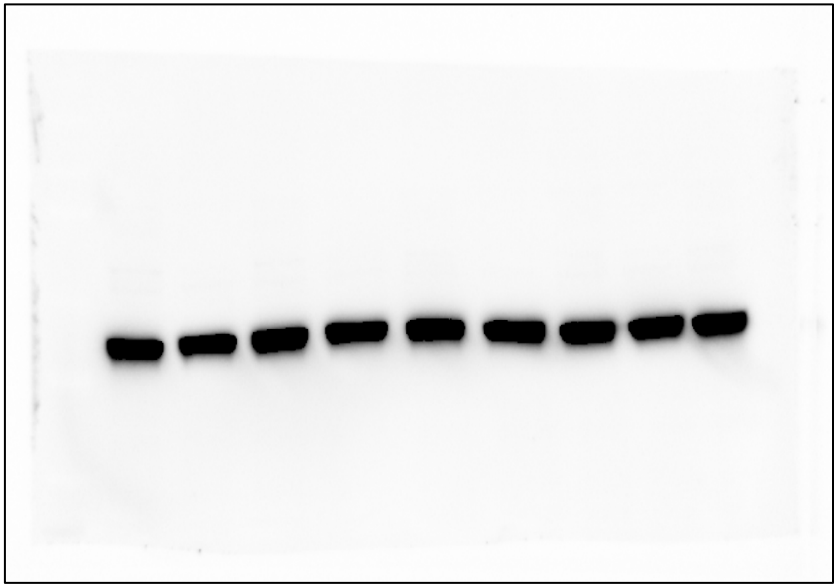

- β-Actin (13E5) Rabbit mAb (HRP Conjugate)
- #5125
- Monoclonal
- Company: Cell Signaling

L= ladder
C=control samples (vehicle)
E= experimental samples (AAS)

S2-C

GnRH (45 KD) β-actin (45 KD)

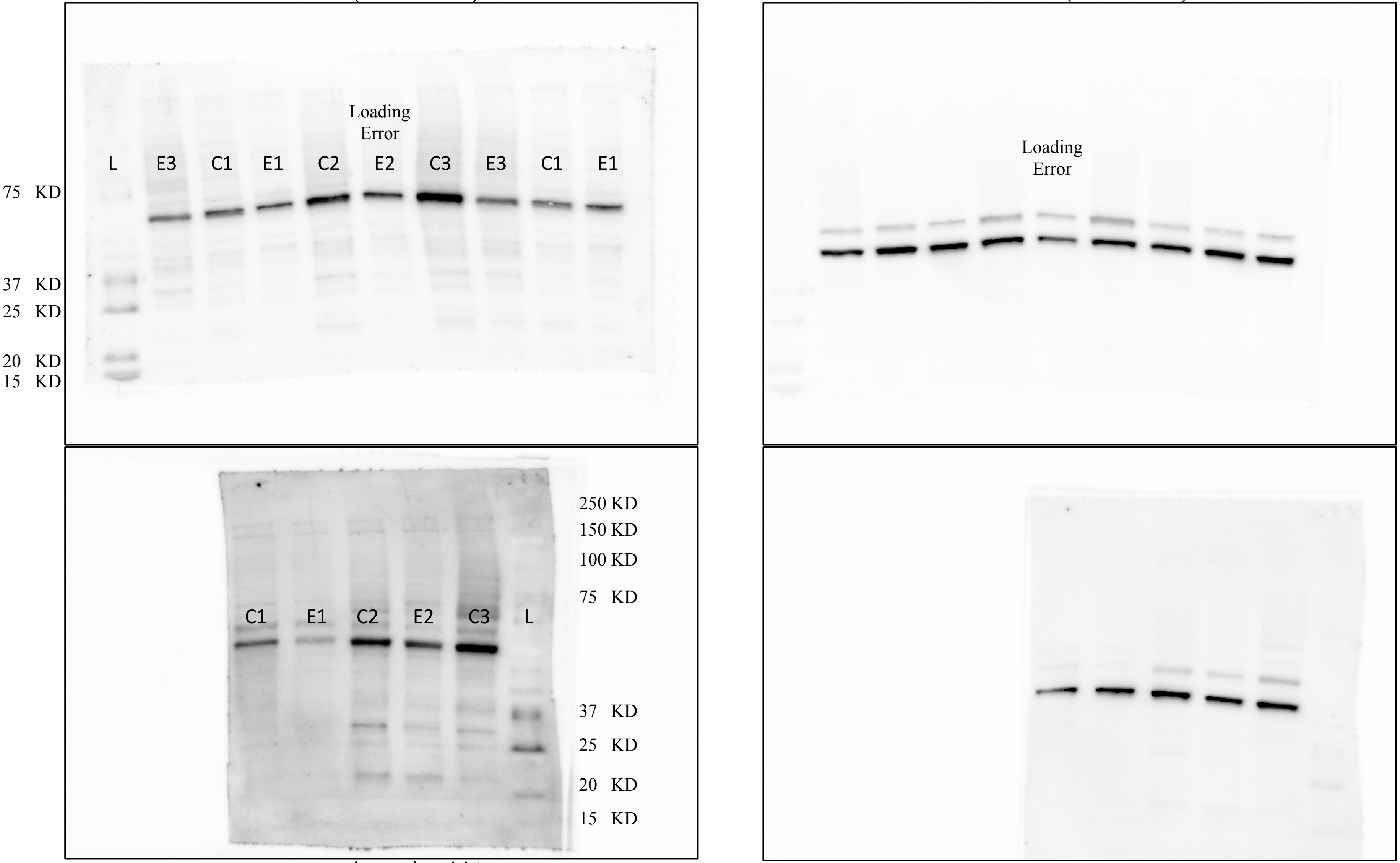

- GnRH 1 (FL-92) Rabbit
- Sc- 20941; Polyclonal
- Company: Santa Cruz

- β-Actin (13E5) Rabbit mAb (HRP Conjugate)
- #5125; Monoclonal
- Company: Cell Signaling

L= ladder
C=control samples (vehicle)
E= experimental samples (AAS)

S2-D

p-ERK 1/2 (44/42 KD)

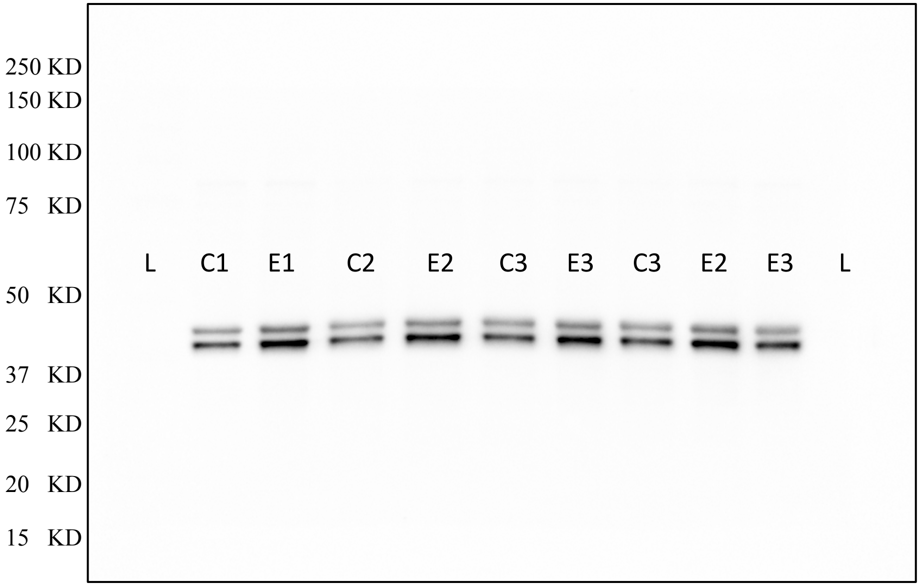

β-actin (45 KD)

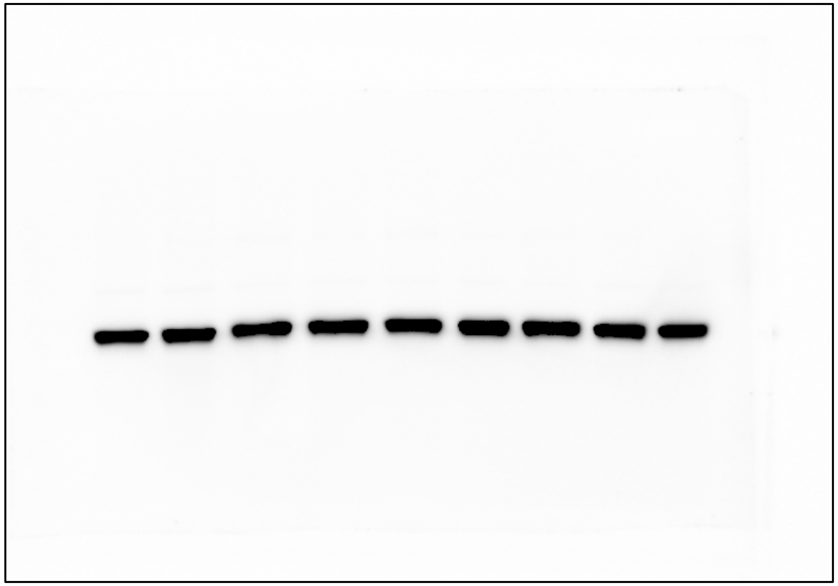

- p-p44/42 MAPK (Erk1/2) (Thr202/Tyr204) (D13.14.4E) XP Rabbit mAb
- # 4370
- Monoclonal
- Company: Cell Signaling

- β-Actin (13E5) Rabbit mAb (HRP Conjugate)
- #5125
- Monoclonal
- Company: Cell Signaling

L= ladder
C=control samples (vehicle)
E= experimental samples (AAS)

S2-E

p-p38 (43 KD)

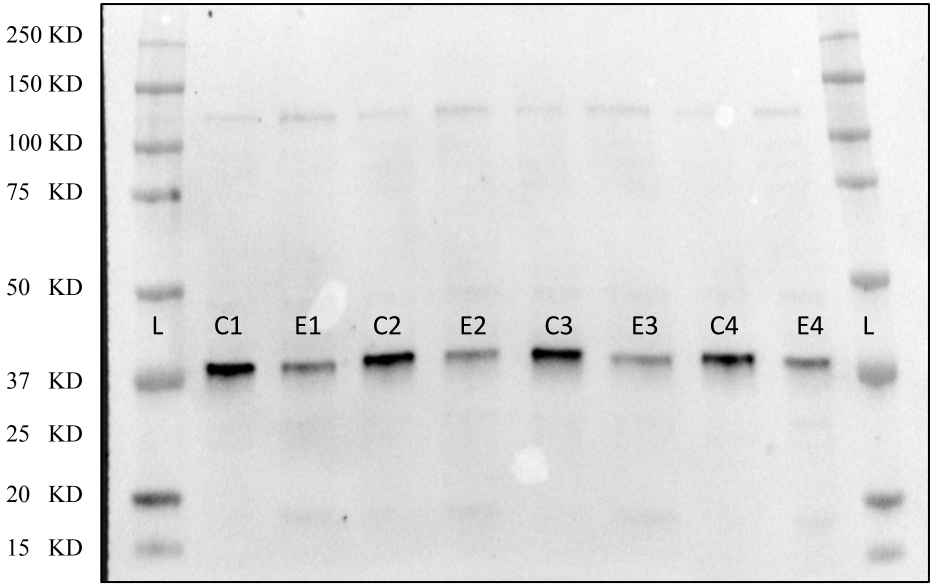

- p-p38 MAPK (Thr180/Tyr182) (D3F9) XP Rabbit mAb
- # 4511
- Monoclonal
- Company: Cell Signaling

β-actin (45 KD)

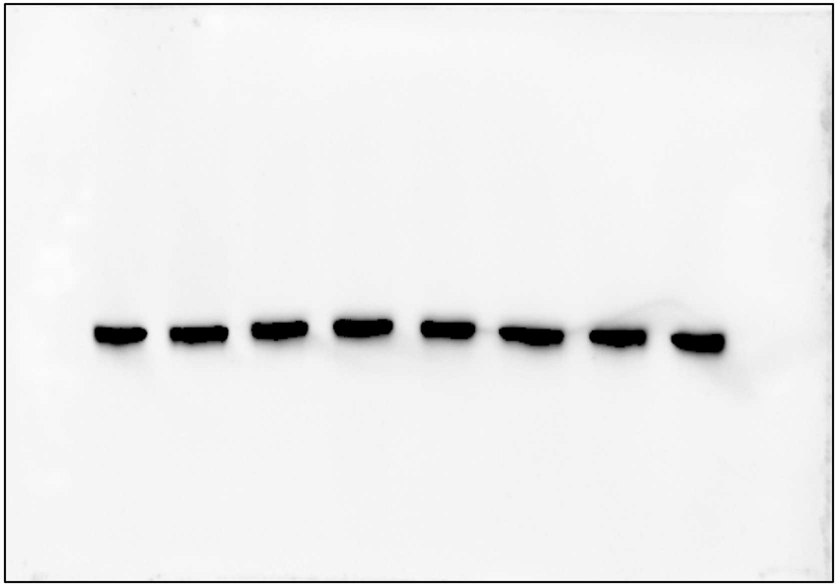

- β-Actin (13E5) Rabbit mAb (HRP Conjugate)
- #5125
- Monoclonal
- Company: Cell Signaling

L= ladder
C=control samples (vehicle)
E= experimental samples (AAS)
